# Towards universal access to skilled birth attendance: the process of transforming the role of traditional birth attendants in Rural China

**DOI:** 10.1186/s12884-016-0854-7

**Published:** 2016-03-21

**Authors:** Hong Jiang, Xu Qian, Lili Chen, Jian Li, Erin Escobar, Mary Story, Shenglan Tang

**Affiliations:** Global Health Institute, Fudan University, Mailbox 175, No. 138 Yixueyuan Road, Shanghai, 200032 China; School of Public Health, Fudan University, Mailbox 175, No. 138 Yixueyuan Road, Shanghai, China; Key Laboratory of Public Health Safety, Ministry of Education, Fudan University, Mailbox 175, No. 138 Yixueyuan Road, Shanghai, China; Maternal and Child Health Hospital, Guangxi Autonomous Region, 225 Xinyang Road, Nanning, 530003 China; Shanghai Municipal Center for Disease Control and Prevention, 1380 Western Zhongshan Road, Shanghai, 200336 China; Duke Global Health Institute, Duke University, 310 Trent Drive, Durham, NC 27710 USA

**Keywords:** Skilled birth attendance, Traditional birth attendant, Institution-based childbirth, Maternal mortality

## Abstract

**Background:**

Institution-based childbirth, with the ultimate goal of universal access to skilled birth attendance (SBA), has been selected as a key strategy to reduce the maternal mortality rate in many developing countries. However, the question of how to engage traditional birth attendants (TBAs) in the advocacy campaign for SBA poses a number of challenges. This paper aims to demonstrate how TBAs in rural regions of China have been integrated into the health system under a policy of institutional delivery.

**Methods:**

Research was conducted through literature and document reviews and individual in-depth interviews with stakeholders of the safe motherhood program in rural Guangxi Zhuang Autonomous Region, China. A total of 33 individual interviews were conducted with regional and local politicians, policy makers, health managers, health providers, civil society members, village cadres for women affairs, former TBAs, village maternal health workers, mothers and their mother-in-laws.

**Results:**

Since 1998, TBA’s traditional role of providing in-home care during childbirth has been restructured and their social role has been strengthened in rural Guangxi. TBAs were redesigned to function as the linkage between women and the health system. A new policy in 1999 shifted the role of TBAs to village maternal health workers whose responsibilities were mainly to promote perinatal care and institution-based delivery of pregnant women. This successful transformation involved engaging with government and other actors, training TBAs for their new role, and providing incentives and sanctions for human resources management.

**Conclusions:**

The China experience of transforming the role of TBAs in Guangxi rural area is an example of successfully engaging TBAs in promoting institution-based childbirth.

## Background

In 1987 the World Bank cosponsored the Safe Motherhood Conference in Nairobi which launched the Safe Motherhood Initiative (SMI). It spurred tremendous attention on reducing maternal mortality globally [1]. Following the SMI launch, the 1990 World Summit for Children set the target for reducing by 50 % the maternal mortality ratio (MMR) by the year 2000 [2]. Subsequently, the Millennium Development Goals (MDGs), approved in 2000, incorporated a target (5a) to reduce the MMR by 75 % between 1990 and 2015, and increase the proportion of births attended by skilled health personnel (skilled birth attendance, SBA) [3].

Since 1949, China has shown great improvement in almost every health indicator [4]; however, China’s MMR, infant mortality and under age five mortality rate stagnated from the mid-1980s to the 1990s [5, 6], with wide MMR disparities between rural and urban areas. In 1990, the average MMR in rural areas was more than double that of urban areas [6]. The Chinese State Council instituted national action plans in 1992 and 1995, respectively, with the goal of reducing by 50 % the national MMR by the year 2000. However, it was clear that the target of reducing MMR in China could not be reached without effective policy interventions to address the disparity gaps between urban and rural areas. Beginning in the early 1990s, projects supported by international organizations were developed to provide training for Traditional Birth Attendants (TBAs) in rural areas [7]. Nevertheless in 1998, after almost10 years of implementation in China, it was clear that training TBAs without having a strong functioning health system to ensure quality maternal services would not be effective for rapidly and significantly reducing the MMR [8]. This was in consistent with the global evidence years later [9–11]. The presence of a skilled birth attendant at childbirth is important in averting maternal and neonatal mortality and morbidity. Based on a situational analysis, an institution-based childbirth policy for rural areas was selected as the cornerstone of the new national safe motherhood policy across China. In order to promote institutional delivery, with the goal of achieving universal SBA, transforming the role of TBA was the first step. TBAs were encouraged to discontinue home delivery, advocate for institution-based childbirth and assist with maternal healthcare as village maternal health workers.

In 2001, the Chinese State Council renewed the national action plans for a new decade from 2001 to 2010, reinforcing the government’s commitment to lower the MMR in line with the MDGs. Today China has met the MDG 5a target [12, 13]. China’s MMR has decreased from 56.2/100,000 nationally to 23.2/100,000, and in rural areas, from 74.1/100,000 to 23.6/100,000 from 1998 to 2013 [12]. The MMR disparity between urban and rural areas has almost disappeared, with 22.4/100,000 and 23.6/100,000, respectively, in 2013 [12]. This paper aims to describe and discuss the process of transforming the role of TBAs in China’s Guangxi Zhuang Autonomous Region as a case study, to illustrate how government successfully integrated TBAs into the health system in rural regions with the ultimate goal of universal access to SBA.

## Methods

### Study setting

Guangxi Zhuang Autonomous Region is located in Southern China and is organized into 14 prefectures, including 14 cities and 109 counties, with a population of about 47,115,000 in 1997. It is a multi-ethnic mountainous area with a primarily agricultural economic base. The region is a less developed area in China; in 1998, 49 counties were defined at or below national or regional poverty level. There was also large disparity regarding economic status between urban and rural populations. For example, in 2006, the annual per-capita income of rural residents was RMB 2,770.5¥ (about USD $335) compared to RMB 9,898.8¥ (USD $1,193) for urban residents. The MMR of Guangxi was higher than the national average: in 1995 it was 96.0/100,000, and in 1998 86.0/100,000. In one particularly poor county, the MMR was as high as 232.4/100,000 in 1998 [14].

Along with its high MMR, Guangxi’s average rate of institution-based childbirth in rural areas was very low. Home delivery had been a long time traditional custom in many rural areas of Guangxi. In 1998 only about 30 % of childbirth in rural Guangxi took place at health institutions, 60 % were attended at home by TBAs previously trained by United Nations International Children’s Emergency Fund (UNICEF) and World Bank projects, and the remaining births were at home without any assistance or attended by untrained female relatives [8]. Moreover, in 30 poor, remote and mountainous counties with limited transportation it was only 18 % [14]. Although there were health institutions at township and county levels who utilized midwifes, nurses or obstetric doctors for birth attendance, the service utilization was low. The barriers preventing mothers from institutional delivery included low economic status, poor transportation and cultural factors [15].

### Data collection and analysis

Literature and document reviews and individual in-depth interviews were conducted by trained research staff in the school of Public Health, Fudan University, China. The literature and document reviews conducted from 2006 to 2014, consisted of 28 articles and policy documents on safe motherhood program in Guangxi region, with emphasis on transforming the role of TBAs at the beginning of the program in 1998. Thirty-two individual interviews were conducted with stakeholders during March 2007and March 2009, including regional and local politicians, policy makers, health managers, health providers, civil society members, village cadres for women affairs, village maternal health workers, former TBAs, mothers and their mother-in-laws (Table 1). The interviews were carried out with a three phased design. In the first phase from January to March 2007, we had two interviews with two key informants to explore the overall process of the safe motherhood program. From these initial interviews, we were able to gather the names of more key informants. Then in the second phase from April 2007 to March 2008, we improved the interview protocols and conducted 28 interviews. After the preliminary qualitative data analysis of the interviews, we conducted two follow up interviews during July 2008 and March 2009 to clarify information that was unclear. All interviewees were purposefully selected according to their position or role during the safe motherhood program in Guangxi. An additional interview with a key policy maker was conducted in March 2014 to discuss program sustainability.

**Table 1:** The qualitative interviews in Guangxi

Type of respondent	Definition	No. of interviews
Politician	Political figures at regional and county levels	4
Policy-maker/planner	Health public sector officials whose responsibilities include the formulation, development, monitoring and implementation of health policies at region and county level	4
Health manager	Programme managers who focus on policy implementation at county and township level	3
Health staff	Health staff in the township hospital who was at the frontline of service delivery	1
Civil society	Organised entities including professional association and international organization	2
Mothers with kid younger than 3 years old	Women in the rural villages who had childbirth within 3 years	8
Mothers-in-law with grandchild younger than 3 years old	Women in the villages whose daughter-in-law had childbirth within 3 years	2
Village cadre for women affairs	Female cadre in the villages responsible for women affairs	2
Village maternal and child health worker	Village maternal and child health worker who transformed from the former traditional birth attendant	2
Former traditional birth attendant	Women who provided attendance for home delivery in villages but stopped home birth attendance after the implementation of institution-based delivery	5
Total		33

In 2014, one more follow up interview was conducted with a key polic maker who had been interviewed in 2007 to understand the policy sustainability

Semi-structured interview guides were developed to explore the process of implementing the safe motherhood program in Guangxi, including transforming the role of TBAs. After informed consent was obtained, face-to-face interviews were conducted in a private room and recorded using a digital pen. Each interview lasted from 0.5 to 2 h. Two researchers conducted the interviews, one as the interviewer and the other as the note taker. Of the 33 interviews, over half (*n* = 19) were conducted in Mandarin, and the rest (*n* = 14) were conducted in the local dialect with the assistance of local translators. All interviews were immediately transcribed to Microsoft Office Word document. Data were coded using Nvivo 7.0 computer software. Two investigators coded each transcript separately and any differences were discussed and resolved. A content analysis approach was used to categorize the content into themes and yielded the headings and sub-headings used below in the results section [16]. The research was approved by the Institutional Review Board of the School of Public Health, Fudan University, China.

## Results

Based on the findings from the literature and document reviews and the qualitative interviews, we used the following four steps to describe the process used to transform the role of TBAs in rural Guangxi: 1) Planning for new roles of TBAs, 2) Piloting policy implementation, 3) Implementation process, and 4) Responses from the health system and the TBAs on the changes.

### Planning for new roles of TBAs

As described by a policy maker in one of the interviews, before the launch of the regional safe motherhood program in 1998, policy makers in Guangxi conducted a situational analysis of the local context and policy. Given geographic challenges and limited health resources, building facilities to provide SBA near each village or to provide timely and qualified emergency services for home delivery was nearly impossible in many rural villages. The literature and document review also indicated that the main cause of maternal death was hemorrhage during home delivery, which underscored the importance of an efficient referral system and emergent care supported by the health system [8]. As the result, an alternative approach was identified, to shift away from home delivery to delivery in townships or higher-level institutions. Promotion of institution-based childbirth was chosen as the key strategy for reducing the MMR in rural Guangxi.

As a village cadre for women affairs said in an interview,

“Before, women had childbirth at home. Sometimes they just delivered by themselves. Nobody helped her and only the woman herself was in the room. If complications occurred, e.g. hemorrhage, people would not discover this until the woman was found after fainting. Women did not know what hemorrhage was”.

As most home deliveries were assisted by TBAs, the question of how to deal with TBAs was the first challenge in developing the safe motherhood program in Guangxi. At the onset, advocacy and mobilization efforts were needed to raise the awareness of institution-based delivery among villagers. It was feasible to involve TBAs as advocates in the villages given their wide social network within the community. Due to the lack of health facilities in remote areas, rural pregnant women often traveled long distances to seek prenatal or postnatal care, and timely follow up care was very low. Given TBAs’ experience, they were capable of promoting prenatal and postnatal care, or providing postnatal care for non-severe cases. The income earned from home delivery varied in different villages and cultural customs, but was generally low and in some cases families supplemented cash payments with food. Because the earning power for TBAs was low, it seemed feasible and acceptable to consider an alternative role with incentives, as part of the advocacy campaign promoting institutional delivery.

**Fig. 1:**
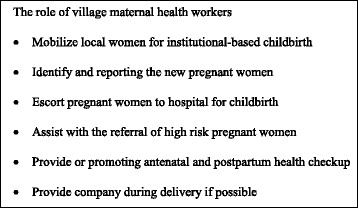
The responsibility village maternal health workers

**Fig. 2:**
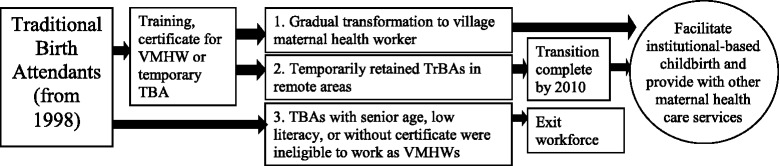
Strategy of transforming the role of Traditional Birth Attendants. (VMHW:village maternal health worker)

A new position, “village maternal health worker,” was planned for TBAs and tasks were shifted to promote perinatal care and institution-based childbirth. They were designed to function at the grassroots level of the rural health service delivery system. The specific responsibility of maternal health care workers included the mobilization of pregnant women for institutional delivery, assisting with home visit for basic care and escorting pregnant women to the hospital for childbirth (Fig. 1). The local health institutions were responsible for providing routine supervision and management and economic compensation for village maternal health workers. In principle, all TBAs working in an area with an available institution-based delivery facility were to assume the responsibilities of the new role or discontinue work as TBAs. In some remote or mountainous areas where institution-based delivery was still a logistical challenge, TBAs could be retained temporarily. TBAs over a certain age, who had low literacy, or being without certification for family birth attendance were not eligible for village maternal health workers; eligibility criteria varied by township (Fig. 2).

### Piloting policy implementation

In 1998 *The Safe Mother and Baby Project* was piloted by the Guangxi Health Bureau in Fucheng township, Beihai city and a minority area Binyang township, Nanning City. TBAs in the area were instructed not to attend births at home. Instead they were asked to assist local township health centers by identifying and reporting new pregnant women, helping to promote prenatal and postnatal health care in township health centers, and escorting the pregnant women to the institutions for childbirth. The pilot indicated that transforming the role of TBAs was both practical and effective. As shown by the document, the rate of institutional delivery in Fucheng township rose from 14.6 % to 85.5 % before and after the pilot [17].

With the increasing rate of institutional delivery in the pilot areas, a key policy document*—Opinion on the Advocacy of Institution-based childbirth, the Establishment of Baby-friendly Health Centers, and the Gradual Transformation of the role of Traditional Birth Attendants in Rural Areas -* was issued by the Guangxi Health Bureau in March 1999. This document formalized the role and regulations for village maternal health workers as noted above. In remote rural areas where it was impossible to adopt health facility delivery due to poor transportation or obstetric human resources, TBAs were retained temporarily before the end of 2010 and received further training.

### Implementation process

#### Advocacy and mobilization

Numerous advocacy and social mobilization activities promoted institution-based childbirth and introduced the new roles of TBAs. Staff in township health centers, related governmental sectors, and members of Non-governmental Organizations such as the Women’s Federation conducted visits to TBAs and villagers to explain the new policy, and facilitated communication between villagers, TBAs and government. As one policymaker said:

“The Working Committee on Children and Women (WCCW, a governmental organization protecting women’s rights and benefits) at county level held meetings with TBAs to convey the new policy and regulation. We told them why they couldn’t attend birth at home any more. We informed them that attending birth at home now was prohibited by the local regulations. I even went to Longan County to give the lecture myself”.

#### Training and management of the new maternal health workers

The county health bureau was responsible for training the new village maternal health workers. Maternal Child Health (MCH) hospitals at different levels and some professional associations provided technical training, which emphasized identifying high-risk pregnancies and assisting with referrals. Following the training, participants were assessed to confirm they met the required standards, and received certification as village maternal health workers, replacing previous certification to perform home delivery service [18]. For TBAs who continued to work in remote areas, the training focused on care during childbirth and referral skills. As a civil society member described:

“The experts of professional association conducted a lot of training for village maternal health workers. They taught them how to escort pregnant women to hospitals for childbirth. Workers also were trained on how to conduct postpartum visit and so on”.

Trained Birth Attendants (TrBAs) who continued to work in remote areas required approval by the county health bureaus. In order to ensure technical competency, the county health bureau required TrBAs to undergo 3 months of additional midwifery training at MCH hospitals. Additionally, TrBAs were required to conduct at least 30 independent deliveries under the supervision of an obstetrician. The retained TrBAs were not permitted to attend deliveries until they received a new “family birth attendant” certificate. Both village maternal health workers and retained TrBAs were registered with the local county health bureau and given technical guidance by the local county or township health centers. Regular meetings were convened for both training and administrative purposes to ensure program success”.

#### Incentive and sanction strategy

**Fig. 3:**
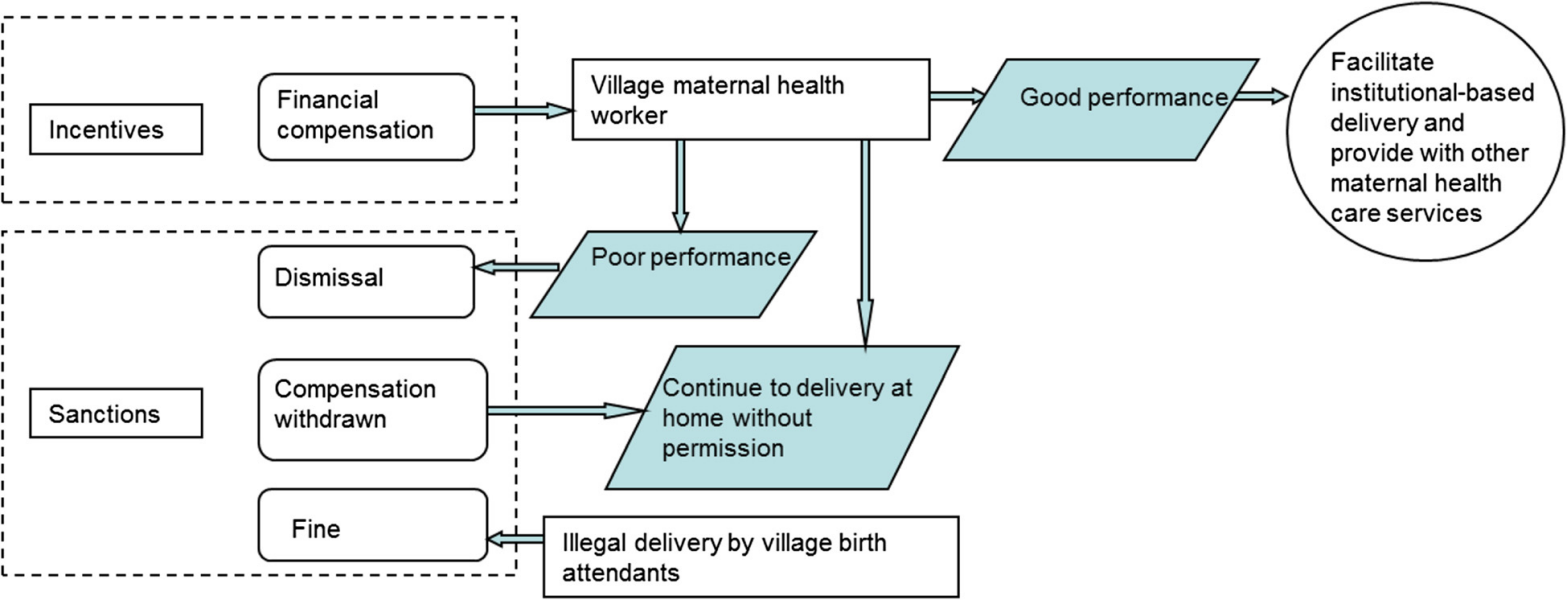
Incentive and sanction mechanisms for transforming the role of Traditional Birth Attendants

Incentives and sanction measures were used to manage the performance of the village maternal health workers (Fig. 3). Financial compensation was used as the main incentive.

As interviewees reflected, village maternal and child health workers could obtain compensation from local health institutions according to each county’s regulations. Generally, they received payment of RMB 20 to 100¥ (USD $2.4 to $12.0) for escorting a pregnant woman to the health institution, and they received approximately RMB 10 to 20¥ (USD $1.2 to $2.4) for providing each postnatal check-up since the transformation was initiated in 1998. Transportation costs were reimbursed. They also received additional payment if they provided other services, such as referral to a health facility for prenatal/postpartum examination. In some areas, workers received a base salary of about RMB 200¥ (USD $24) per month, and obtained bonuses for additional services provided. As a Health Manager said

“The general approach in Guangxi was to pay RMB 20-100¥ to village maternal health workers for provision of prenatal examination for one pregnant woman. But [in our county] we also provided RMB 200¥ per month as a basic salary for each worker. In addition to this base salary, we added RMB 50¥ each time they escorted a pregnant woman to the health facility, with additional compensation for a postpartum examination. Payment was distributed at monthly meetings. Total [monthly] income for each worker was at least RMB 500¥ and some reached RMB 1,000¥.”

A village maternal and child health worker (previously a TBA) said,

“Now, usually I will have the prenatal check-up for pregnant women [in the village]. Then I will take her to hospital for childbirth. After her delivery and return to the village, I will go visit her and conduct a postnatal examination. If she had an episiotomy, I will help remove stitches. The instrument is provided by the village health station. I will get RMB 20¥ from the hospital when I escort a woman, and RMB 10¥ for the postnatal visit”.

The government also implemented a sanction mechanism. If a delivery was attended by uncertified individuals, a warning was issued by the county health bureau or the corresponding institution. The illegal income was confiscated and a fine was levied. According to the management regulation, if the illegal income was above RMB ¥5,000 (USD $602), the amount of the fine should be at least three times but under 5 times of the illegal income. If there was no illegal income or the amount was less than RMB ¥5000 (USD $602), the fine should be above RMB ¥5,000 (USD $602) and less than RMB ¥20,000 (USD $2,409). If a village maternal health worker was found attending a home birth, compensation was withheld.

### Responses from health system and by TBAs on the change

#### Health system in responding to the changes

Key changes were implemented in the health system to promote the uptake of institutional delivery. Obstetric departments and Emergency Obstetric Centers were constructed. Funds were allocated to the health system to improve the obstetric department infrastructure. A greater emphasis was placed on removing financial, geographic, and cultural barriers of institution-based childbirth. Financial subsidies were allocated to incentivize pregnant women to deliver in a health facility. Specific vehicles and boats were provided free to women in areas with poor road conditions. Stretcher action was initiated to send women to health institutions in mountainous areas [19]. To remove cultural barriers, changes were implemented to make the wards warm and inviting, i.e., colorful quilts replaced white bedding which was regarded inauspicious in some ethnic minorities. Civil society organizations were actively involved in training village maternal health workers. Obstetric health human resources grew rapidly: more than 28,000 midwives with at least 3 years of vocational training were recruited and assigned to township health centers by 2007.

Although reforms in health systems occurred along with transforming the role of TBAs, maternal deaths unexpectedly occurred in health facilities due to the inadequate preparation for the rapid increase of institution-based childbirths. As a key policy maker recalled, when the average rate of institutional delivery continuously increased from 60 % in 2000 to 72 % in 2003, the MMR rebounded from 60/100,000 to 69/100,000 (also recored in the document [17]), since many health facilities, such as township health centers, were not well prepared for the upsurge in deliveries. Fortunately this trend reversed in 2004 after continuous efforts were made in health system, for example, more investments for building infrastructure and referral networks, assigning obstetric experts from provincial and municipal areas to rural areas to provide training, emergent obstetric care and supervising routine work.

#### Former TBAs’ reaction to the changes

Given the perceived risk of negative consequences, most TBAs stopped attending home deliveries. As one health manager recalled:

“Most TBAs were cooperative after several advocacy and mobilization attempts since they knew the conditions, and their qualifications and compentences couldn’t match the service demands. They recognized the risk of family delivery. They thought the role transformation could also mean a release for them”.

Because income had been low for TBAs, continuing home deliveries was not attractive. One health manager and one former TBA said respectively:

“They [TBAs] were cooperative; since the quantity of deliveries they attended before transformation was not very much, they earned little from delivery. Additionally, after transformation, they needn’t take any more risk over birth attendance. So, they were not strongly antagonistic towards the policy”.

“The way people paid me [for assistance of home delivery] was flexible. At the beginning, people did not pay for my service; they gave me some eggs and sticky rice instead. Then they paid me around RMB 1 to 2¥ and gradually increased to RMB 10¥ later. Before the institution-based childbirth was advocated here [around 1998], people sometimes gave me around RMB 100¥ [for per home delivery]. But now I did not assist birth at home any more. I support people to go to the hospital for childbirth. I do not need to worry and be stressed anymore. Although I did have income from this, it was not much. I did not depend on this for my living”.

Most TBAs perceived the new role as lower risk, and were able to accept and undertake the new responsibilities after adequate training. A small number of TBAs resisted the transformation but overall discord was low. As one Health Manager noted, “Some individuals were resistant to the policy, but it was not very strong”.

One of the findings was that awareness of institution-based childbirth among villagers improved. As two village women said,

“People used to have childbirth at home. Nobody told us to go to the hospital [for childbirth]. I had my first child born at home. Now the service is very good at the hospital, they [health staff] gave my baby a bath. They also provided a free pail and towel for the baby’s bath”.

“The cost of institution-based delivery is acceptable. I think it is safer to have childbirth in a hospital than at home. If an emergency occurs, they [health staff] can rescue. No difficulty now… I spent RMB 400¥ in the hospital and got reimbursed RMB 150¥ after I returned home.”

It was estimated there were a total of about 30,000 TBAs in 1997 in Guangxi. By 2006, over 50 % of TBAs had been converted to village maternal health workers, about 35 % left their TBA’s position, and only less than 2,000TBAs were retained [20]. With the synergistic effect of other enabling factors in Guangxi, institutional delivery increased from 46 % in 1998 to 90 % in 2006 and 99.8 % in 2013. The MMR decreased from 86.0/100,000 in 1998 to 27.2/100,000 in 2006, and further to 14.2/100,000 in 2013 [6, 17]. By the end of 2013, there were no TBAs in rural Guangxi. All former TBAs, even those in remote areas, now have been changed to maternal health workers or no longer do home delivery.

## Discussion

Institution-based childbirth, with the ultimate goal of universal access to SBA is a key strategy to reduce maternal mortality rate in many low income countries. The experience of transforming the role of TBAs in a rural area of China (Guangxi) is an example of successfully engaging TBAs in a new role to advocate for SBA in areas where institution-based childbirth was low.

Currently, the way of promoting SBA varies throughout the world. Practices in different countries have shown TBAs can play a helpful role no matter which policy of the SBA was adopted. In Malaysia where there has been a dramatic decline in the MMR, TBAs were encouraged to provide supporting services such as massage, working in partnership with midwives directly employed by the government [21]. In Sri Lanka, where there also has been a marked MMR reduction, public health midwives in the community assist with the health unit and serve as a linkage to the institution-based delivery service [22]. The strategy applied in Guangxi, as well as in Malaysia and Sri Lanka, actively involved original TBAs through their community role transformation rather than excluding them from the health system.

The argument of whether and how to integrate TBAs into the health system still exists [23–25]. Our study demonstrated the approach of incorporating TBAs into the health system, and serving as a linkage between the health system and pregnant women. TBAs were designed to act as the linkage instead of providing intrapartum care, which was consistent with international advocacy of SBA [9, 26]. On one side, this linkage promoted the policy dissemination to rural areas and extended the care from health system to women. On the other side, it largely helped rural women who were usually isolated from outside society to uptake the service provided by the health system. One study indicated that even in the context of institutional delivery, TBAs were still culturally and interpersonally needed by women and should be incorporated to the health system [23]. The planning of transforming the role of TBAs in Guangxi included similar or even greater potential earning power for the village maternal and child health worker than their original job and helped to eliminate or alleviate some resistance by the TBAs. During the process of transforming the role of TBAs, advocacy efforts at the grass-root level helped communities with a long history of home delivery to understand the policy. Logistical considerations, as well as, cultural and religious customs, were considered by the health institutions. Inclusion criteria and management mechanisms for village maternal health workers were established as key components to ensure service quality.

The case of transforming the role of TBAs in Guangxi also indicated lessons learned. There must be sufficient preparation within the health system prior to the transformation. The preparation should be comprehensive, including training of health human resources, building infrastructure, improvement of service quality, and establishment of referral channels and quality referral centers. While institution-based childbirth was advocated and promoted, removing financial, geographic and cultural barriers must be taken into account since these factors affect women’s access and use of care [27, 28].

In the case of Guangxi, the village maternal health worker was financially supported by county hospitals or township health centers. The workers’ income was linked with the economic status of those institutions, which weakened the sustainability of the policy. Sustainable financing mechanisms need to be developed. Furthermore, the planning process of transforming the role of TBAs did not actively involve the villagers and TBAs, which could have increased barriers for the transformation process. The involvement of all the stakeholders should have taken place in the early stage of the transformation.

One of the weaknesses of this study is that the Guangxi Safe Mother and Baby Project to reduce MMR did not solely focus on transforming the role of TBAs. Therefore, the independent effect of transforming TBAs in reducing MMRs cannot be established. Importantly, while transformation of the role of TBAs is only one of the steps, it is the first step needed to scale up institutional delivery.

## Conclusion

The experience of transforming the role of TBAs in Guangxi, China is an example of successfully engaging TBAs to assume a new role to promote institution-based childbirth. Through policy development, engaging all government departments and civil society actors, training TBAs for their new role, and providing incentives and sanctions for human resources management, the transformation was shown to be feasible, acceptable and successful. The strategies used in this paper can serve as an example for countries and regions facing similar challenges.

## Abbreviations

MCH: Maternal Child Health
MDGs: The Millennium Development Goals
MMR: Maternal mortality
SBA: Skilled birth attendance
SMI: Safe Motherhood Initiative
TBAs: Traditional birth attendants
TrBAs: Trained Birth Attendants
UNICEF: United Nations International Children’s Emergency Fund
WCCW: The Working Committee on Children and Women
